# Tianma Gouteng Yin, a Traditional Chinese Medicine decoction, exerts neuroprotective effects in animal and cellular models of Parkinson’s disease

**DOI:** 10.1038/srep16862

**Published:** 2015-11-18

**Authors:** Liang-Feng Liu, Ju-Xian Song, Jia-Hong Lu, Ying-Yu Huang, Yu Zeng, Lei-Lei Chen, Siva Sundara Kumar Durairajan, Quan-Bin Han, Min Li

**Affiliations:** 1 School of Chinese Medicine, Hong Kong Baptist University, Hong Kong; 2 Mr. & Mrs. Ko Chi-Ming Centre for Parkinson’s Disease Research, Hong Kong Baptist University, Hong Kong; 3 State Key Laboratory of Quality Research in Chinese Medicine, Institute of Chinese Medical Sciences, University of Macau, Macao

## Abstract

Tianma Gouteng Yin (TGY) is a traditional Chinese medicine (TCM) decoction widely used to treat symptoms associated with typical Parkinson’s disease (PD). In this study, the neuroprotective effects of water extract of TGY were tested on rotenone-intoxicated and human α-synuclein transgenic *Drosophila* PD models. In addition, the neuroprotective effect of TGY was also evaluated in the human dopaminergic neuroblastoma SH-SY5Y cell line treated with rotenone and the rotenone intoxicated hemi-parkinsonian rats. In rotenone-induced PD models, TGY improved survival rate, alleviated impaired locomotor function of *Drosophila,* mitigated the loss of dopaminergic neurons in hemi-parkinsonian rats and alleviated apoptotic cell death in SH-SY5Y cells; in α-synuclein transgenic *Drosophila*, TGY reduced the level of α-synuclein and prevented degeneration of dopaminergic neurons. Conclusively, TGY is neuroprotective in PD models both *in vivo* and *in vitro*.

Parkinson’s disease (PD) is the second most common age-dependent, late-onset neurodegenerative disease in humans^1^,^2^. Numerous factors including environmental and genetic factors contribute to the onset and progression of PD. One environmental neurotoxin implicated in the pathogenesis of PD is the commonly used insecticide rotenone. Due to its lipophilic character, rotenone readily passes across the blood brain barrier. It is suspected to be a mitochondrial complex I inhibitor that inhibits the transfer of electrons from iron-sulfur centers to ubiquinone and thereby elicits PD-like symptoms^2^. With regard to genetic factors in the development of PD, SNCA (α-synuclein) has been identified as a causative gene in familial forms of PD^3^. Through genome-wide association studies (GWAS), SNCA has been further identified as one of the risk factors for rare monogenic forms of PD^4^. The neuropathological characteristic of patients with SNCA mutations is widespread α-synuclein deposits in neurons and glial cells^5^.

Currently, both environmental neurotoxin-intoxicated and transgenic PD models have been developed^6^. Among these models, the fruit fly *Drosophila melanogaster* has emerged as a frequently used model organism for studying PD-related neurodegeneration. Both environmental neurotoxin models and transgenic models have been successfully established in *Drosophila* to replicate key pathological features of PD^7^,^8^.

Tianma Gouteng Yin (TGY) is one of the most famous traditional Chinese medicine (TCM) decoctions commonly prescribed by TCM practitioners to treat PD-like symptoms such as tremor and paralysis^1^. However, conclusive experimental evidence to support TGY’s clinical application in the treatment of PD is lacking. In this study, rotenone-intoxicated and α-synuclein overexpressed *Drosophila* models and rotenone-induced hemiparkinsonian rats were employed as *in vivo* models to study the anti-PD activity of TGY. The underlying neuroprotective mechanism was also analyzed *in vitro* using rotenone-intoxicated SH-SY5Y cells.

## Results

### Preparation and LC/MS analysis of TGY

**Figure 1:**
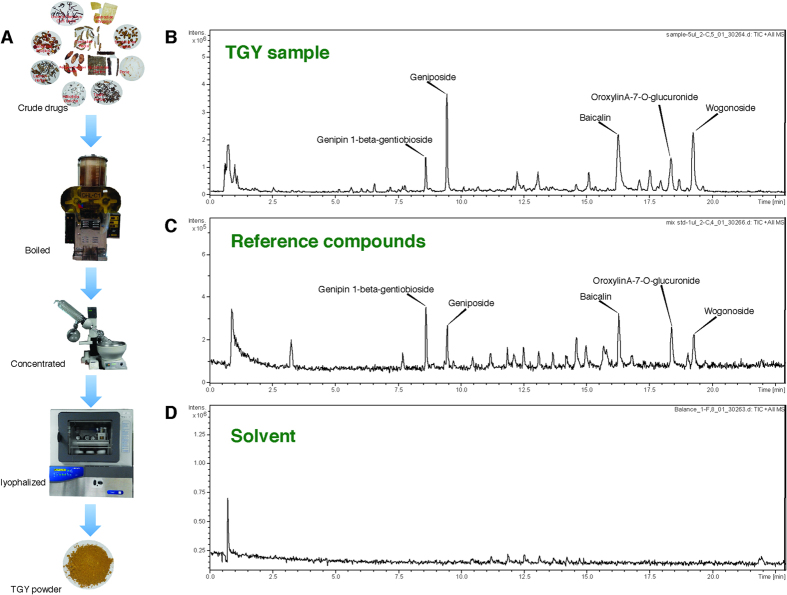
Preparation and qualitative analysis of TGY. (**A**) Crude materials of TGY were pulverized and boiled with tap water. The water extract was then concentrated and subjected to lyophalisation. The yields were powdered and store in –20 °C; the powder of TGY was then analyzed by HPLC-ESI-qTOF-MS. (The photos were taken by L. F. L.)The MS spectrum in positive ion mode of TGY was obtained (**B**) and some of the constituents were denoted by reference compounds (**C**) in contrast to the solvent baseline (**D**).

Similar to most TCM formulas, in clinical application, TGY is prepared in decoction. Controlled quality of TGY is pivotal to its pharmacological activity and repeatability of the experiments. In order to keep the consistent quality of TGY as it is used in clinical practice, TGY was extracted by water and prepared scrupulously. The preparation procedure is summarized in Fig. 1A. Quality analysis of TGY was performed with a method we developed (to be published elsewhere) using ultra high-performance liquid chromatography (UPLC) coupled with quadrupole-tandem time-of-flight mass spectrometry (Q-TOF-MS). The TGY powder used in this study, prepared with this method, was analyzed. As shown in Fig. 1B, the MS spectra reveals the presence of genipin-1-beta-gentiobioside, geniposide, baicalin, oroxylin-7-O-glucuronide and wogonoside, by comparison with reference compounds (Fig. 1C) and matrix solvent (Fig. 1D).

### TGY antagonized rotenone toxicity in *Drosophila*

**Figure 2:**
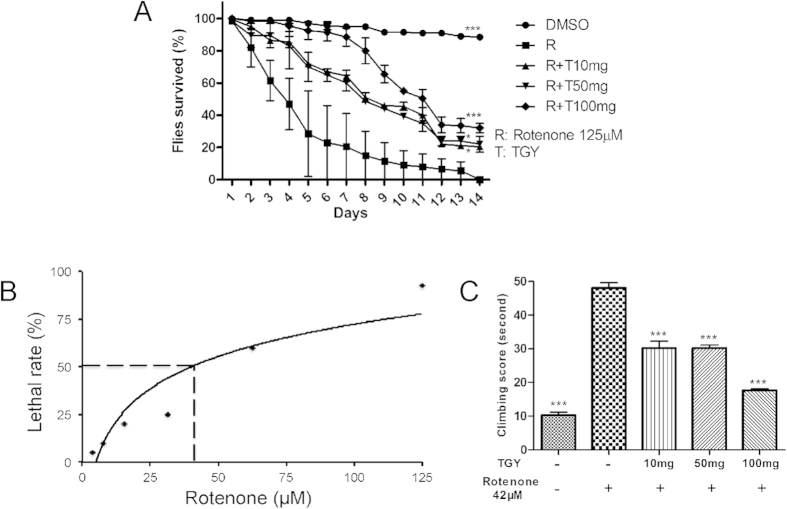
TGY protected flies from rotenone intoxication. (**A**) Survival curves of the flies treated with rotenone with and without TGY were plotted. TGY increased the survival of the flies dose-dependently. (**B**) The LD50 of rotenone on *w*^*1118*^ was tested. (**C**) Climbing assay was performed to test the motor function of the flies. TGY alleviated the impairment of rotenone on motor function. *p < 0.05, **p < 0.01, and ***p < 0.001 compared with rotenone treated flies. Results were obtained from three independent replicates.

Rotenone has been reported as an environmental neurotoxin inducing PD-like phenotypes in *D. melanogaster*^7^. Based on the preliminary toxicity assay, male w1118 flies receiving 125 μM rotenone died within 14 days (Fig. 2A) and the median lethal dose (LD_50_) of rotenone was calculated to be 42 μM (Fig. 2B). To test whether TGY is protective against rotenone intoxication, we fed the flies with rotenone and different dosages of TGY concurrently. As shown in Fig. 2A, almost 80% of the flies died within 7 days and all died by the end of the experiment. However, the flies fed with TGY in addition to rotenone showed better survival rates. More than 80% of the flies treated with 100 mg of TGY were alive on the 7^th^ day and no less than 30% of flies survived at the end of the experiment. In addition, TGY significantly alleviated motor dysfunction caused by rotenone intoxication (Fig. 2C). In comparison with the normal flies, the flies intoxicated by the sublethal dosage of rotenone (42 μM) for 14 days exerted distinct motor function loss: most of the flies were unable to climb and stayed at the bottom of the vials. This phenotype was quantified by climbing assay. The result (Fig. 2C) shows that, in the presence of rotenone, the flies commonly could not reach the finishing line within the time limit (50 s). In contrast, in the absence of rotenone, only 15 s were needed for the flies to reach the finishing line. The climbing defect induced by rotenone was partially rescued by TGY treatment. The climbing ability of the flies treated with 100 mg of TGY was significantly improved as indicated by the improvement in the climbing score (~20 s).

### TGY reduced α-synuclein level and suppressed neurotoxicity in transgenic *Drosophila*

**Figure 3:**
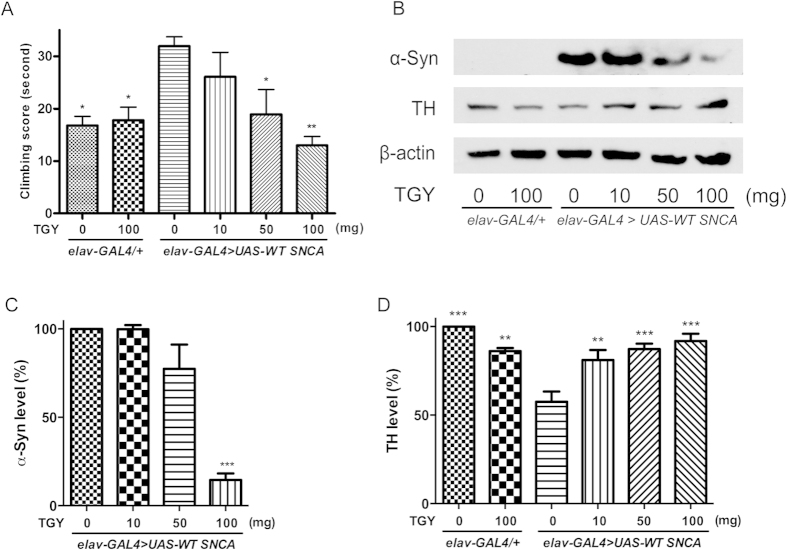
TGY alleviates the neurotoxicity induced by α-synuclein pan-neural over expression. WT-α-synuclein was over expressed under the neuronal specific driver *elav-GAL4*. The flies were treated with TGY for 30 days. It was found that (**A**) TGY protects the flies from the impairment of motor function induced by WT-α-synuclein. (**B–D**) The level of WT-α-synuclein was decreased while the level of Tyrosine hydroxylase (TH), the marker protein of dopaminergic neuron, increased accordingly. *p < 0.05, **p < 0.01, and ***p < 0.001 compared with model flies without treatment. Results were obtained from three independent replicates.

Abnormal α-synuclein accumulation is one of the major culprits causing PD. *Drosophila* overexpressing α-synuclein recapitulates the key features of human PD^8^,^9^, such as loss of dopaminergic neurons, neuronal inclusion containing α-synuclein and locomotor defects^8^,^9^,^10^. These *Drosophila* PD models have been successfully utilized in testing the activities of small molecules and gene function^11^,^12^. In the current study, we tested TGY on flies overexpressing α-synuclein. Wild type human α-synuclein (WT-α-synuclein) was pan-neurally overexpressed by *elav-GAL4*. The 30-day-old α-synuclein transgenic flies exhibited locomotor dysfunction as indicated by the climbing test scores. The aged α-synuclein transgenic flies took more than 30 s to reach the finishing line while the normal control flies (elav-GAL4/+) spent less than 20 s (Fig. 3A). In addition to the behavioral results, the level of the marker protein of dopaminergic neurons, tyrosine hydroxylase (TH), was decreased strikingly in the presence of α-synuclein (Fig. 3B). As a result of α-synuclein toxicity, the flies lost 40% of TH in comparison with normal control flies (Fig. 3D). In contrast, TGY dose-dependently alleviated locomotor dysfunction (Fig. 3A). The flies treated with 100 mg of TGY spent less than 20 s to reach the finishing line, which was similar to the normal controls. As shown in the typical blots, TGY prominently reduced the α-synuclein protein levels (Fig. 3B,C) in the brains of the flies. Meanwhile, the flies treated with TGY preserved more TH then than untreated α-synuclein overexpressed flies (Fig. 3B,D). The results indicate that TGY alleviated the neurotoxicity triggered by α-synuclein overexpression and subsequently protected the dopaminergic neurons in the brains of the flies.

### TGY reduced α-synuclein induced dopaminergic neuron loss in transgenic *Drosophila*

**Figure 4:**
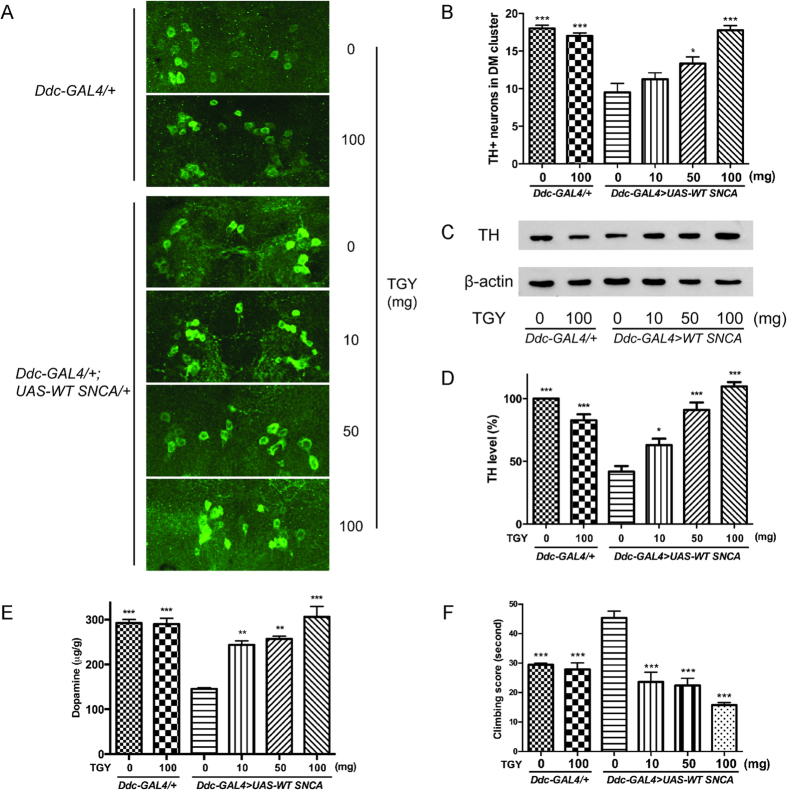
TGY rescued the loss of dopaminergic neurons caused by WT-α-synuclein over expression. WT-α-synuclein was over expressed by the dopaminergic neuron specific driver *Ddc-GAL4*. Flies were treated with TGY for 30 days. (**A**) Brains were dissected and immunostained with TH antibody to visualize the dopaminergic neurons. (**B**) The number of TH positive neurons in dorsal medial region was calculated (Five brains per group of each experiment). (**C**) The lysate of the brains were subjected to western blotting analysis and (**D**) the TH levels of each group were compared by densitometry analysis. To verify the histology and western blotting results, (**E**) the brains of the flies were extracted for LC/MS analysis to determine the dopamine levels. The results were consistent with (**F**) the data of climbing assay of each group of flies. *p < 0.05, **p < 0.01, and ***p < 0.001 compared with model flies without treatment. Results were obtained from three independent replicates.

To further verify whether TGY could specifically counteract dopaminergic neuron degeneration caused by overexpression of α-synuclein, *Ddc-GAL4,* a dopaminergic neuron-specific GAL4 driver, was employed to overexpress human WT-α-synuclein exclusively in the flies’ dopaminergic neurons. Full spectrum analyses of the dopaminergic neuron systems of the flies’ brains were performed. The flies were fed with TGY for 30 days and then decapitated. The heads were immunostained with TH through whole mount assay. TH-positive neurons in dorsomedial (DM) dopaminergic neuronal cluster of *Drosophila* brains were shown and counted (Fig. 4A,B). The number of TH positive neurons suggested that TGY prevented dopaminergic neuron loss caused by WT-α-synuclein. The immunoblot data (Fig. 4C,D) showed that TGY maintained the TH level in transgenic PD flies, which was consistent with the immunohistochemistry analysis data. To further confirm these findings, dopamine levels in the flies’ brains were determined using LC/MS analysis. The result (Fig. 4E) showed that TGY preserved dopamine levels in the brains of transgenic *Drosophila.* The data of dopamine analysis and the TH protein level data obtained in immunoblot analyses together prove that TGY prevents dopaminergic neuron loss resulting from α-synuclein toxicity. In the climbing assay, TGY exhibited a protective effect in comparison with the untreated model flies (Fig. 4F), which was also consistent with the previous findings.

### TGY alleviated rotenone-induced apoptotic cell death in SH-SY5Y cells

**Figure 5:**
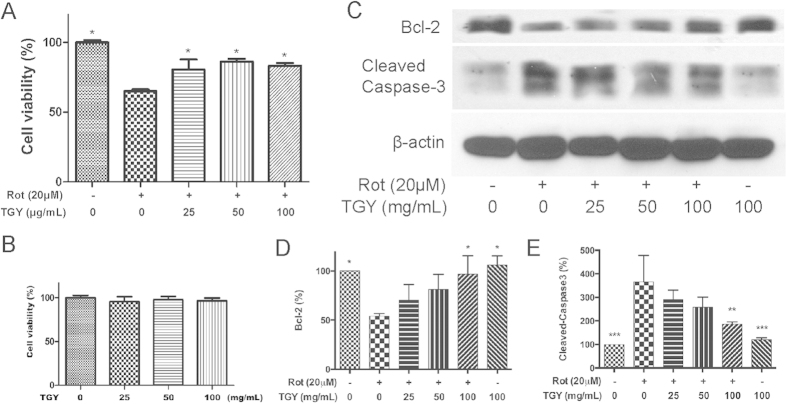
TGY prevented apoptosis induced by rotenone on SH-SY5Y cells. It was found that (**A**) Rotenone induced cell death was counteracted by TGY. (**B**) TGY extract is safe to the SH-SY5Y cells ≤100 μg/mL. (**C**) Apoptotic cell death, as indicated by unregulated cleaved Caspase-3 and down regulated pro-survival protein Bcl-2, caused by rotenone treatment, was alleviated by TGY. (**D,E**) The Bcl-2 and cleaved-Caspase 3 levels in different groups were compared by densitometry analysis. *p < 0.05, **p < 0.01, ***p < 0.001 compared with rotenone-intoxicated cells without treatment. Results were obtained from three independent replicates.

To further investigate the neuroprotective mechanisms of TGY, we tested the anti-apoptosis effect of TGY on human dopaminergic SH-SY5Y cells by adding rotenone to induce apoptotic cell death^13^. SH-SY5Y cells were treated with 20 μM of rotenone and co-treated with different dosages of TGY for 24 hrs. The cell viability was tested by MTT assay. Result of the MTT assay indicates that TGY prevented cell death induced by rotenone (Fig. 5A). In immunoblot analysis, the pro-survival protein Bcl-2 and the apoptosis marker cleaved-caspase3 were detected. TGY preserved the level of Bcl-2 and reduced the cleavage of caspase3, which indicates TGY alleviated rotenone-induced apoptosis (Fig. 5C–E).

### TGY mitigated dopaminergic neurons loss in hemi-parkinsonism rats

**Figure 6:**
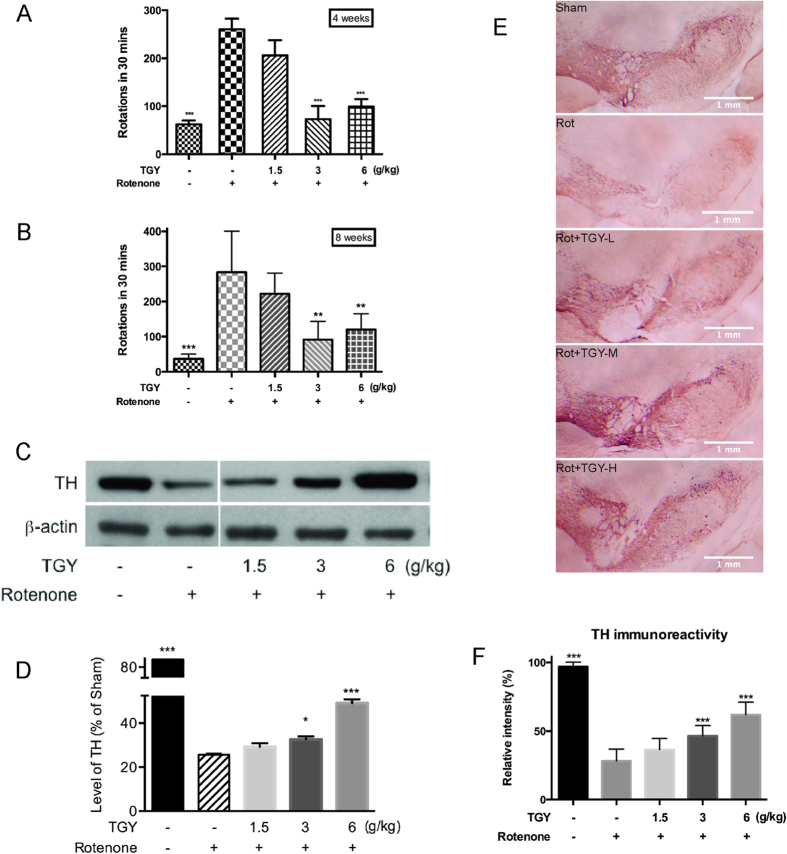
TGY mitigated the rotenone induced dopaminergic neurons loss in rats. (**A**) TGY significantly prevented rotational behaviors provoked by APO in 4 weeks after rotenone intoxication, and (**B**) avoided exacerbation in 8 weeks post intoxication. (**C,D**) TGY alleviated the decrease of TH in SNpc as indicated by the immunoblot result (n = 3) and confirmed by (**E,F**) TH DAB staining data (n = 3). *p < 0.05, **p < 0.01, ***p < 0.001 compared with rotenone-intoxicated cells without treatment.

The neuroprotective effect of TGY was further examined in mammalian PD model. The rotenone-intoxicated hemi-parkinsonian rats were used in the study. Rats stereotaxically infused with rotenone to ventral tegmental area (VTA) and substantia

nigra pars compacta (SNpc) increasingly displayed rotational behavior that provoked by apomorphine (APO) injection time dependently^14^. Four weeks after infused with rotenone, the rats displayed strong rotational behavior (~10 circles/min, Fig. 6A) after challenged with APO. However, the rats receiving TGY significantly reduced the APO-provoked rotations. The APO-provoked rotations in rotenone-intoxicated rats receiving 3 and 6 g/kg TGY were reduced by nearly 60% compared with those rotenone-intoxicated rats with vehicle treatment. The rotational behavior was further tested at the end of treatment (8 weeks). As shown in Fig. 6B, the rotenone intoxicated rats showed intensified APO-provoked rotations while TGY treatment notably prevented the deterioration. This data indicated TGY antagonize the damaged dopaminergic system function caused by rotenone infusion. To further verify whether TGY could prevent dopaminergic neuron loss, the ipsilateral part of the SNpc of the rats were harvested and subjected to immunoblot analysis. The marker protein of dopaminergic neuron, tyrosine hydroxylase (TH), was probed. The typical blot was shown in Fig. 6C. As shown in the figure, rotenone infusion drastically reduced the level of TH, which indicated rotenone resulted in severe degeneration of dopaminergic neurons. TGY alleviated the degeneration dose dependently. As indicated by the densitometry analysis of the blots (Fig. 6D), almost ~80% of the dopaminergic neurons were loss 8 weeks after rotenone intoxication, while the rats treated with 6 g/kg TGY preserved about 60% of the dopaminergic neurons. The results were further confirmed by histological examination of brain tissues. The typical TH 3,3′-diaminobenzidine (DAB) staining photos of SNpc from each group were shown in Fig. 6E. The integrated intensity of TH immunostaining was examined by densitometry analysis. In SNpc, the results were quite consistent with the immunoblot result, the immunoreactivity of rotenone intoxicated rats showed ~80% loss TGY treated rats showed ~60% of immunoreactivity compared to that of the sham rats (Fig. 6F).

## Discussion

In the present study the anti-PD activity of TGY with controlled quality was evaluated in *in vivo* and *in vitro* models of PD. Treatment with TGY significantly suppressed rotenone-induced death and locomotor dysfunction of *Drosophila.* Feeding with TGY notably reduced α-synuclein protein levels and comparatively prevented loss of dopaminergic neurons in the brains of *Drosophila.* The neuroprotective effect of TGY was further confirmed on hemi-parkinsonian rats. In stereotaxic rotenone intoxication rats, TGY significantly improved the behavior and mitigated dopaminergic neurons loss. Meanwhile, the anti-apoptosis activity of TGY was also verified in SH-SY5Y cells. These results provide experimental evidences supporting the application of TGY for the treatment of PD.

TGY is traditionally used in the form of decoction. Hence, in this study, we used water to extract TGY following a standardized preparation. In the TGY water extract, flavonoids and iridoids, such as baicalin and geniposide, are the most abundant small molecules (Fig. 1B). These compounds have been shown to have anti-inflammatory, anti-oxidative and anti-apoptotic activities^15^,^16^,^17^,^18^. All of the compounds in TGY constitute the chemical foundation of its pharmacological activity. How the components of TGY exert anti-PD activity deserves further investigation.

In this study, the insecticide rotenone was introduced to established *in vivo* and *in vitro* PD models. Rotenone was reported as an inhibitor of mitochondrial complex I and an inducer of dopaminergic lesion^19^. Through treating *Drosophila* with lethal and sublethal doses of rotenone, a PD model was established to evaluate the anti-PD activity of TGY (Fig. 2). In using this model, we found that special attention must be paid to the variability in susceptivity to rotenone of different *Drosophila* strains. In the present study, we performed the experiments all with the same *w*^*1118*^ strain^20^. We also treated SH-SY5Y cells^21^ with rotenone to induce apoptotic cell death. Mild anti-apoptotic cell death activity of TGY was observed (Fig. 5), indicating anti-apoptotic mechanism plays an important role in the neuroprotective activity of TGY. The neuroprotective effect of TGY in terms of preventing dopaminergic neuron loss caused by rotenone was further confirmed in hemi-parkinsonian rat (Fig. 6). In this model, direct infusion of rotenone into the SNpc and VTA resulted in loss of dopaminergic neurons in these two brain regions. We pretreated the rats prior to rotenone infusion to evaluate the preventive effect of TGY. The data obtained on this hemi-parkinsonian rat model confirmed our finding in *Drosophila.* In summary, TGY treatment could mitigate the toxicity of rotenone.

In PD, α-synuclein is the major pathogenic protein. The mutations in this protein accelerate its aggregation then contribute to the onset and progress of PD^22^. Thus, reducing the formation and increasing the clearance is a reasonable strategy for developing a therapy for PD. *Drosophila* is a widely used model organism in studying neurodegenerative diseases^23^. By using the GAL4/upstream activating sequence (UAS) system^24^, ectopic overexpression of human α-synuclein in the brain of *Drosophila* was established as a genetic PD model^11^. In the current study, we utilized *elav-GAL4*^25^ to drive pan-neural expression of α-synuclein. Overexpressed α-synuclein impaired dopaminergic neurons as indicated by decreased TH level in immunoblot analysis and defective ability in the climbing assay. TGY treatment reduced α-synuclein and rescued the dopaminergic neurons (Fig. 3). In this result, the flies receiving 10 mg TGY did not reduce the level of α-synuclein but alleviated the decrease of TH. This result indicated TGY exhibited neuroprotectivity that prevented the loss of dopaminergic neurons without affecting the level of α-synuclein at this dosage. In other words, TGY protected against α-synuclein-induced neurotoxicity not only through degrading α-synuclein. Another issue is that *elav-GAL4* drive the expression of the transgene in the whole neural system. Thus, all of the neurons were affected by α-synuclein. Hence we used a dopaminergic neuron-specific GAL4 driver, *Ddc-GAL4,* to exclusively express α-synuclein in the dopaminergic neurons of adult flies^26^. We implemented a broad spectrum of analyses focusing on dopaminergic neurons. The results demonstrate that TGY protected dopaminergic neurons from α-synuclein impairment. From these two *Drosophila* PD models, we provided sufficient evidence that TGY decreases α-synuclein in the neurons and thereby, prevents dopaminergic neuron loss consequently.

In conclusion, our data indicate that TGY is neuroprotective in PD models. This corroborates its ongoing use in traditional Chinese medicine to treat PD-like symptoms. Evidence that it works immediately initiates a flood of further inquiries as to how it works. For instance, how does TGY impact the misfolded and oligomerized α-synuclein that is thought to be more toxic than the monomer^27^? How does TGY regulate abnormalities in lysosome or proteasome function that is important in clearance of α-synuclein^28^? In addition, since PD is a disease affecting central nervous system, it would be very important to understand the brain permeability of TGY phytochemicals and their pharmacokinetics features. Further experiments are needed to answer all of these questions.

## Materials and Methods

### Reagents and antibodies

Rotenone (R8875), sodium dodecyl sulfate (SDS, L3771), N, N-dimethylformamide (DMF, D4551), Dimethyl sulfoxide (DMSO, D8418), Phenylmethanesulfonyl fluoride (PMSF, 78830), Ethylenediaminetetraacetic acid (EDTA, E9884), Ethylene glycol-bis(2-aminoethylether)-*N,N,N′,N′*-tetraacetic acid (EGTA, E3889) and dopamine hydrochloride (H8502) were purchased from Sigma-Aldrich. Goat anti-mouse (115-035-003) and goat anti-rabbit (111-035-003) antibodies were purchased from Jackson ImmunoResearch Laboratories, INC. Alexa Fluor 488 conjugated goat anti-rabbit secondary antibody (A-11008) and 3-(4,5-dimethylthiazol-2-yl)-2,5-diphenyltetrazolium bromide (MTT, M-6494) were purchased from Thermo Fisher Scientific Inc. Anti-tyrosine hydroxylase antibody (anti-TH, AB152) was purchased from Merck Millipore. Anti-α-synuclein (610787) was purchased from BD Transduction Laboratories. Anti-β-actin (sc-47778) was purchased from Santa Cruz Biotechnology.

### Preparation of TGY extract and fly food

Dry materials of the ingredients in proportions as listed here were pulverized. Roots and rhizomes were cut into small pieces prior to being pulverized. One (human) dose of TGY is: *Gastrodiae Rhizoma (Tianma)* 9 g, *Uncaria Ramulus Cum Uncis (Gouteng)* 12 g, *Haliotidis Concha (Shijueming)* 18 g, *Gardeniae Fructus (Zhizi)* 9 g, *Scutellariae Radix (Huangqin)* 9 g, *Cyathulae Radix (Chuanniuxi)* 12 g, *Eucommiae Cortex (Duzhong)* 9 g, *Leonuri Herba (Yimucao)* 9 g, *Taxilli Herba (Sangjisheng)* 9 g, *Polygoni Multiflori Caulis (Shouwuteng)* 9 g, *Poria (Fuling)* 9 g. After pulverizing, the powder was steeped with threefold tap water (v/w) for 0.5 h, boiled for 1 h, and then filtered. The residue was extracted with tap water another two times. The filtrate was concentrated by rotary evaporation under vacuum in a 60 °C water bath. The concentrated extract was frozen in liquid nitrogen and finally subjected to lyophilisation under vacuum of 105 × 10^−3^ mbar and −40 °C. Final yield was powdered and then stored at −20 °C. All medical materials were purchased from Hong Kong Baptist University Mr. & Mrs. Chan Hon Yin Chinese Medicine Specialty Clinic and Good Clinical Practice Centre. The voucher specimens were deposited at the School of Chinese Medicine, Hong Kong Baptist University, Hong Kong, China. TGY and/or rotenone was mixed thoroughly with rehydrated instant *Drosophila* medium (Carolina, USA). The medium was renewed every 3 days.

### LC/MS analysis

Agilent 1290 UPLC system (Agilent Technologies), equipped with an auto sampler, a binary pump and a thermostatted column compartment, was used for chromatographic analysis. The samples were separated on ACQUITY UPLC BEH C18 column (2.1 mm × 100 nm, 1.7 μm) at 40 °C. The mobile phase was composed of 0.1% formic acid in water (A) and 0.1% formic acid in ACN (B). Programmed gradient elution was performed as follow: 0–3 min, 2% B; 3–9 min, 2–12% B; 9–24 min, 12–32% B; 24–29 min, 32–75% B; 29–29.1 min, 75–100% B; 29.1–32 min, 100% B; 32.1–35 min, 100-2%B. The flow rate was 0.4 mL/min. Agilent 6540 Q-TOF mass spectrometer (Agilent Technologies) equipped with a jet stream electrospray (ESI) ion source was utilized to acquire MS and MS/MS data in positive ion mode. Data acquisition was managed by MassHunter B.03 software (Agilent Technologies). The working parameters were as follow: nebulizing gas (N_2_) flow rate, 8.0 L/min; nebulizing gas temperature, 300 °C; jet stream gas flow, 9 L/min; sheath gas temperature, 350 °C; nebulizer, 45 psi; capillary, 3000 V; skimmer, 65 V; Oct RFV, 600 V; fragment voltage, 150 V. Mass spectrum was documented with mass range at 100–1700 m/z of all mass peaks.

### *Drosophila* culture and strains

Fly stocks were raised at 25 °C on standard cornmeal medium, under 50% ~ 70% relative humidity with 12 hrs dark-light cycle. The following *Drosophila* strains were obtained from Bloomington *Drosophila* Stock Center and used in the study: *w*^*1118*^ as wild type; *elav-GAL4,* which drive transgene expression in all neurons; *Ddc-GAL4* that drive transgene expression in dopaminergic neurons.

### Housing and husbandry of rats

Inbred adult male Sprague-Dawley rats (220–260 g) from the Laboratory Animal Service Centre, The Chinese University of Hong Kong were used. The rats were housed under standard conditions with 12 hrs light/dark cycles and constant room temperature of 22 ± 2 °C and 60 ± 5%. They were fed with standard lab diet and sterilized water *ad libitum*. The rats were accommodated to the environment for one week prior to further experiments. All experimental protocols were approved by the Hong Kong Baptist University Committee on the Use of Human & Animal Subjects in Teaching and Research and conducted in accordance with the guidelines for the use of experimental animals of Hong Kong Baptist University.

### Survival assay

*w*^*1118*^ flies emerged within three days were collected and aged for three days in standard cornmeal. The flies were then transferred to vials containing freshly prepared instant medium (Carolina, Formula 4–24) and dosed as indicated every three days. The number of dead flies was counted every day. The percentage of flies that remained alive at the end of the experiment was calculated based on the starting number of flies for each treatment group.

### Climbing assay

Climbing assay was performed as described^29^ with minor modification. Briefly, groups of ten flies were transferred into 18 cm transparent plastic tubes 1 hr prior to the assay for environmental acclimatization. A finishing line at 10 cm from the bottom of the tubes was marked. The tubes were tapped to send flies to the bottoms of the tubes. The climbing time was recorded when at least five flies had passed the finishing line. When flies took more than 50 s to climb above the finishing line, the climbing time was recorded as 50 s. Five trials were performed for each group. The experiment was repeated at least three times. The mean climbing times were calculated and plotted as climbing scores.

### Stereotaxic rotenone intoxication hemi-parkinsonisom rat model and TGY treatment

Total 40 rats were used, the rats were randomly allocated to 5 groups: Sham group (Infused with 1 μl Dimethyl sulfoxide). The rest of the rats were infused with rotenone and then assigned into the other 4 groups including Rotenone group (Model group, received vehicle treatment); TGY low dosage group (TGY-L, received 1.5 g/kg TGY extract); TGY middle dosage group (TGY-M, received 3 g/kg TGY extract) and TGY high dosage group (TGY-H, received 6 g/kg TGY extract). All TGY groups received two weeks pretreatment prior to rotenone intoxication and two months post treatment. The stereotaxic rotenone infusion was performed as previously described with minor modification^14^. Briefly, the rats were anesthetized with intraperitoneal injection of chloral hydrate (3.0% w/v in normal saline, 1 ml/100 g b.d.w.) and then placed and fastened on the stereotaxic frame (Stoelting, Wood Dale, USA). Rotenone was dissolved in DMSO with a final concentration of 3 μg/μl, 1 μl of this solution was infused into the right VTA (AP: 5.3 mm; ML: 0.9 mm; DV: 8.0 mm) at a flow rate of 0.2 μl/min. The needle was hold for extra five minutes for the thorough diffusion of rotenone. After that, the needle was withdrawn slowly. Then rotenone was infused into the right SNc (AP: 5.3 mm; ML: 2.0 mm; DV: 8.0 mm) at a flow rate of 0.2 μl/min, with additional five-minute needle retention. After operation, rats received 200 k Unit Penicillin by subcutaneous injection per day for total three days to prevent postsurgical infection.

### Apomorphine induced rotation

All groups of the rats were tested for APO-provoked rotations at the fourth and eighth week after drug administration. The rats were placed in an opaque plastic cylinder (diameter = 30 cm) for 10 min acclimation, and then challenged with APO (3 mg/kg) via intraperitoneal injection. The following rotational behavior was then recorded for 35 min. The rotations were counted from 6 to 35 min. The rats were placed back into their cages after rotational testing.

### Immunohistochemical analysis

For *Drosophila* whole brain staining, protocol was followed as reported with minor modification^30^. Flies were anesthetized under CO_2_ and then decapitated with forceps. Heads were transferred to 1.5 ml microcentrifuge tubes containing fixative (4% paraformaldehyde, 0.3% Triton X-100). Tubes stayed on ice for 3 hrs to make sure that fly heads were submerged in fixative. The brains were dissected under stereomicroscope, fixed at room temperature for 30 min and incubated with 0.3% PBST (0.3% Triton X-100, 1 × PBS) three times on a rotator, each time 20 min; then blocked for 1 hr at room temperature with blocking buffer (5% normal goat serum, 1 × PBS, 0.1% Triton X-100), then incubated with anti-TH (1:200 dilute in blocking buffer) for 36 hrs at 4 °C on a rotator. The primary antibody was removed and the brains were washed with 0.3% PBST three times on a rotator, each time 20 min, then incubated in goat anti-rabbit Alexa 488(1:200 dilute in blocking buffer). Next, brains were washed with 0.3% PBST three times on a rotator, each time 20 min, and finally mounted with FluorSave™ Reagent (Merck). Mounted brain samples were analyzed under a confocal microscope (Leica, TCS SP5, Germany). For the rat samples, coronal sections (30 μm thickness) were cut throughout the striatum (from 1.0 mm to 2.0 mm caudal to the bregma) by using Shandon Cryotome SME Cryostat (Ramsey, Minnesota, USA). The sections were placed on the slides, air-dried, and then treated with 1% H_2_O_2_ for 10 min to quenched the endogenous peroxidase activity. After washed with 0.3% Triton-X100, the sections were blocked with diluted horse serum for 30 min at room temperature. After blocking, the sections were incubated with TH antibody (Millipore, AB152, 1:500) for 24 hrs at 4 °C. The sections were incubated with ABC® Elite (VECTOR, Burlingame, USA) secondary biotinylated goat anti-rabbit IgG for 30 min at room temperature. Finally the sections were incubated with DAB for 5 min. The sections were then air-dried and mounted with Leica mounting buffer. The slides were then observed under motic SMZ-171 microscope (Hong Kong, China).

### Immunoblot analysis

For *Drosophila* samples, immunoblot analyses were carried out as previously described^9^ with minor adjustments. Twenty fly heads were homogenized in lysis buffer (50 mM Tris, 1%NP-40, 0.35% sodium deoxycholate, 150 mM NaCl, 1 mM EDTA, 1 mM EGTA, 1 mM PMSF, pH7.4) and lysed on ice for 20 min. The supernatants were collected after centrifugation (14,000 rpm, 10 min, 4 °C) and denatured with 5 × Laemmli sample buffer and then separated on 12% SDS-PAGE gels. The protein on the gels were then transferred to Polyvinylidene fluoride (PVDF) membranes (GE Healthcare, RPN303F) and processed for immunoblotting. Membranes were blocked with 5% nonfat milk and probed with primary and secondary antibodies. The bands were developed with an ECL kit (Pierce, 32106). The density of the bands was quantified by densitometry (ImageJ, NIH). For cell samples, lysis buffer was added and the cells were scraped off, transferred to microcentrifuge tubes and then lysed on ice for 30 min. The remaining steps were the same as those used for the fly samples. For the rat brain samples, the ipsilateral striatum was dissected and homogenized with phosphate buffered saline (PBS) containing 1 mM PMSF (1 mg brain added 10 μl PBS). The striatum was homogenized by sonication (120 w, 60 Hz, 2 s per cycle, total 25 blasts, BRANSON, USA) performed on ice. Then the lysate was centrifuged at the speed of 14,000 rpm for 30 min at 4 °C. The remaining steps were the same as those used for the fly samples.

### Dopamine quantification

*Drosophila* brain dopamine levels were analyzed by HPLC/MS method^31^. After treatment, 10 heads of flies from each group were homogenized in 100 μL pre-chilled 0.1 M formic acid. The samples were centrifuged at 13,000 rpm, 4 °C for 10 min. The supernatants were collected and freeze-dried, and then reconstituted with 100 μL ice-cold methanol; they were then centrifuged at 13,000 rpm, 4 °C for 10 min. The supernatants were collected and subjected to LC/MS analysis. Dopamine levels were measured with triple quadrupole LC/MS system (Agilent 6460) equipped with a ZIC pHILIC 150 × 2.1 mm, 5 μm column (Merck).

### Cell culture and cell viability assay

Human neuroblastoma cells SH-SY5Y were maintained in Dulbecco’s Modified Eagle’s medium (DMEM, Invitrogen, 12800017), supplemented with 15% Fetal bovine serum (FBS, Invitrogen, 10099141). MTT assay was employed to evaluate cell viability as previously described^32^. SH-SY5Y cells were seeded in 96-well plates (5,000 cells/well). After 24 hrs, different treatments were performed. In the assay, 20 μM of rotenone was used to induce cytotoxicity^33^. After treatment, the media were removed and phenol red-free DMEM containing MTT (final concentration 0.5 mg/mL) was added, and cells were incubated for 4 hrs. The MTT solution was removed, and the cell crystals were dissolved using 100 μL of 20% SDS in 50% DMF. The intensity was measured using a plate reader at 570 nm with the reference of 620 nm.

### Statistics analysis

Each experiment was repeated at least three times unless indicated. The data was expressed as means ± SD. One-way ANOVA followed by Dunnett’s multiple comparison tests were conducted. All statistics analyses were performed in GraphPad Prism6 (GraphPad Software, Inc.).

## Additional Information

**How to cite this article**: Liu, L.-F. *et al.* Tianma Gouteng Yin, a Traditional Chinese Medicine decoction, exerts neuroprotective effects in animal and cellular models of Parkinson's disease. *Sci. Rep.* **5**, 16862; doi: 10.1038/srep16862 (2015).
